# Association between homocysteine and blood pressure in the NHANES 2003–2006: the mediating role of Vitamin C

**DOI:** 10.3389/fnut.2024.1379096

**Published:** 2024-05-03

**Authors:** Peng Wu, Juan Ma, Shaobin Yang, Hailiang Wu, Xueping Ma, Dapeng Chen, Shaobin Jia, Ning Yan

**Affiliations:** ^1^First Clinical College, Ningxia Medical University, Yinchuan, China; ^**2**^Heart Centre & Department of Cardiovascular Diseases, General Hospital of Ningxia Medical University, Yinchuan, China; ^**3**^Ningxia Medical University, Yinchuan, China; ^**4**^Institute of Medical Sciences, General Hospital of Ningxia Medical University, Yinchuan, China

**Keywords:** homocysteine, Vitamin C, blood pressure, mediation effect, national health and nutrition examination survey

## Abstract

**Background:**

The yearly escalation in hypertension prevalence signifies a noteworthy public health challenge. Adhering to a nutritious diet is crucial for enhancing the quality of life among individuals managing hypertension. However, the relationship between vitamin C and hypertension, as well as homocysteine, remains unclear.

**Objective:**

The primary aim of this investigation was to scrutinize the potential mediating role of Vitamin C in the association between homocysteine levels and blood pressure, utilizing data extracted from the National Health and Nutrition Examination Survey (NHANES) database.

**Methods:**

A total of 7,327 participants from the NHANES 2003–2006 were enrolled in this cross-sectional survey. The main information was obtained using homocysteine, Vitamin C, systolic blood pressure (SBP) and diastolic blood pressure (DBP). Correlation analysis was used to assess the correlation between homocysteine, SBP, DBP and vitamin C. Linear regression analysis was utilized to determine the β value (β) along with its 95% confidence intervals (CIs). Mediation analysis was performed to investigate whether the relationship between homocysteine and blood pressure was mediated by Vitamin C, and to quantify the extent to which Vitamin C contributed to this association.

**Results:**

The results manifested that the homocysteine was positively associated with SBP (*r* = 0.24, *p* < 0.001) and DBP (*r* = 0.03, *p* < 0.05), while negatively correlated with Vitamin C (*r* = −0.008, *p* < 0.001). Vitamin C was found to be negatively associated with SBP (*r* = −0.03, *p* < 0.05) and DBP (*r* = 0.11, *p* < 0.001). Mediation effect analysis revealed that a partial mediation (indirect effect: 0.0247[0.0108–0.0455], *p* < 0.001) role accounting for 11.5% of total effect, among homocysteine and SBP. However, the mediating effect of Vitamin C between homocysteine and DBP was not statistically significant.

**Conclusion:**

Hypertension patients should pay attention to homocysteine and Vitamin C level. What is more, hypertension patients ought to formulate interventions for Vitamin C supplementation as well as homocysteine reduce strategies to lower blood pressure.

## 1 Introduction

Hypertension, characterized by elevated arterial blood pressure, is a well-known cause of ischemic heart disease, stroke, other cardiovascular conditions and contributes to over 10 million deaths globally annually (1, 2). According to the World Health Organization (WHO), the current global estimate for hypertension patients exceeds 1.39 billion (3). Given its widespread occurrence and substantial risk of mortality, hypertension constitutes a pivotal contributor to the global burden of disease (4). Hypertension is a key risk factor contributing to both the incidence and fatality of cardiovascular diseases. Therefore, effectively managing hypertension is crucial for reducing major adverse cardiovascular and cerebrovascular events (5).

Many researches have demonstrated that several risks are linked to hypertension, including obesity, consumption of alcoholic beverages, sodium, homocysteine and vitamin intake (6–8). Homocysteine, an amino acid containing sulfur, is produced via the metabolic pathways of methionine, an essential amino acid obtained from dietary proteins (9). Previous studies have indicated that heightened levels of plasma homocysteine induce oxidative stress and endothelial dysfunction. Consequently, this cascade effect results in vasoconstriction, increased arterial stiffness, and impaired vasodilation of nitric oxide, ultimately contributing to elevated blood pressure (10, 11).

Additionally, insights from various studies have indicated a connection between homocysteine and Vitamin C. Vitamin C levels showed an inverse correlation with homocysteine levels (12, 13).

**Figure 1:**
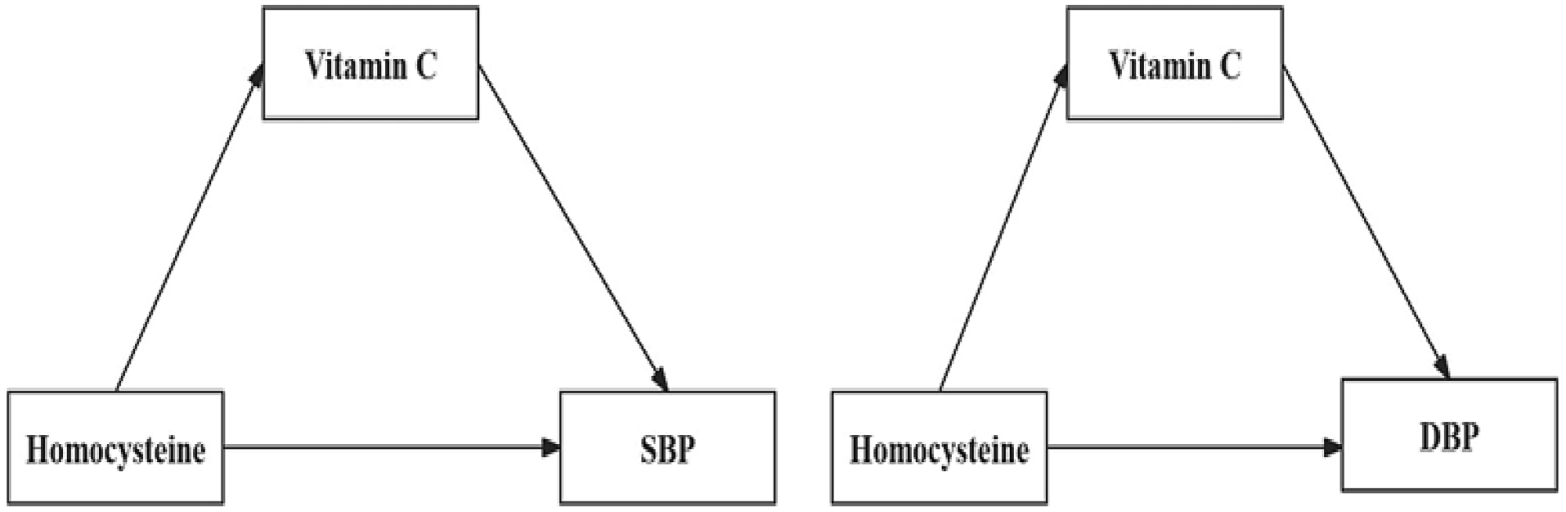
Mediating effect of Vitamin C between Homocysteine and SBP/DBP.

The elevation of homocysteine levels may lead to increased oxidative stress, thereby escalating the demand for intracellular vitamin C, consequently resulting in its depletion (14, 15).Vitamin C, acting as a robust antioxidant, has the ability to neutralize free radicals, reducing oxidative stress and cellular damage. A deficiency in Vitamin C may lead to a weakened antioxidant defense system, making endothelial cells of blood vessels more susceptible to oxidative damage and, consequently, affecting vascular function. Vitamin C decline may itself be a risk factor for hypertension (16). Hence, our hypothesis was that connection between homocysteine and blood pressure could potentially be mediated by the influence of Vitamin C (as shown in Figure 1). Consequently, the aims of our study encompassed an examination of the association between homocysteine and blood pressure, along with the evaluation of the potential mediating role of Vitamin C in this relationship.

## 2 Objects and methods

### 2.1 Study population

**Figure 2:**
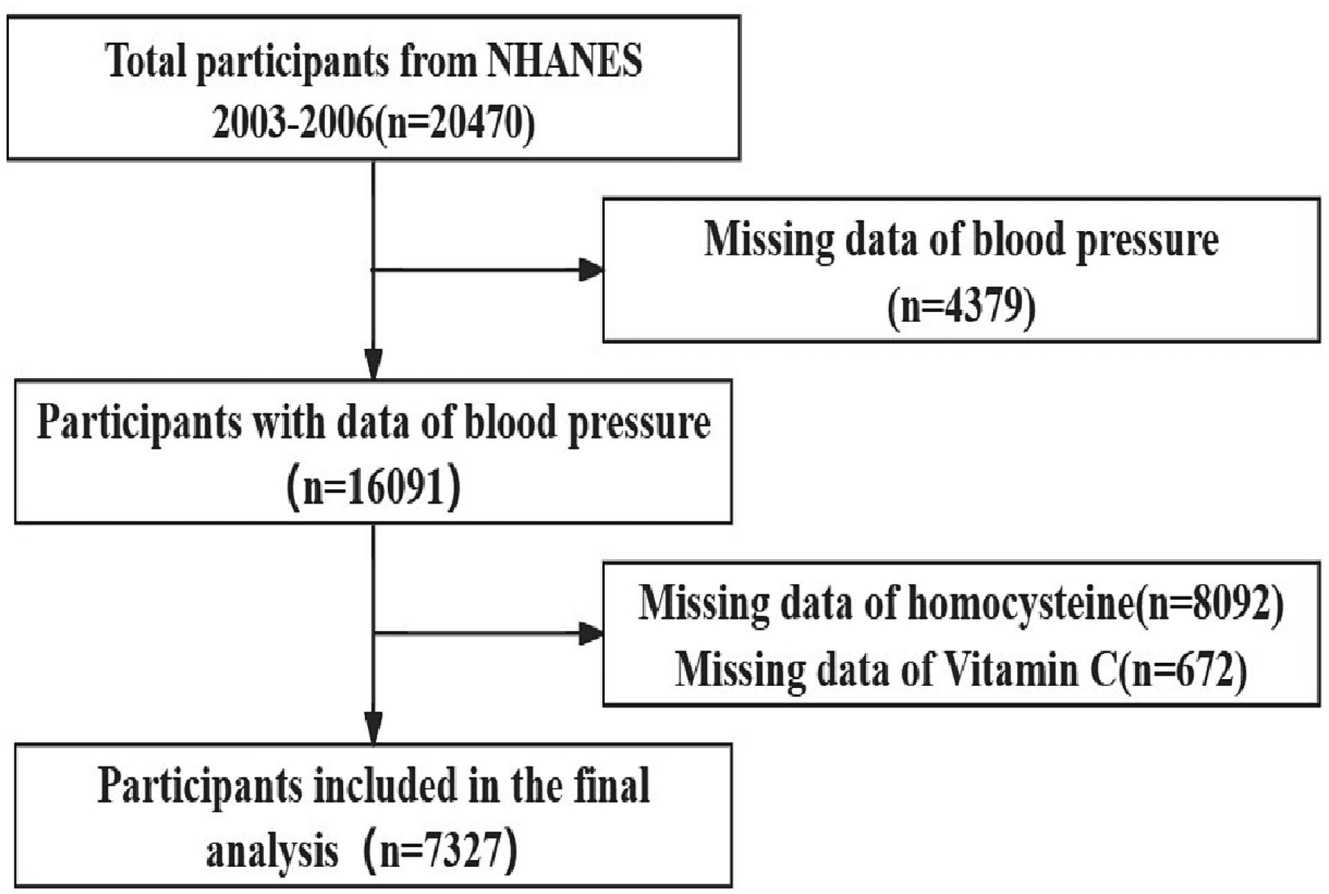
Flowchart of participants included in the analysis.

The NHANES is a cyclic, nationally representative cross-sectional survey targeting non-institutionalized civilian populations in the United States. Conducted biennially by the National Center for Health Statistics (NCHS), its principal objective is the comprehensive assessment of health and nutritional statuses across the United States (17). The population for this cross-sectional study was 20470 subjects from the NHANES (2003–2006). Participants were excluded due to missing data on homocysteine (*n* = 8092), missing data on Vitamin C (*n* = 672) or missing data of blood pressure (*n* = 4379). The final analysis encompassed a total of 7327 individuals (Figure 2). The data utilized in this study were sourced from a publicly available database (accessed on 11 May 2023).^1^ Approval for this study was granted by the National Center for Health Statistics Research Ethics Review, and explicit written consent was secured from all participants.

### 2.2 Plasma homocysteine measurement

For NHANES 2003–2006, homocysteine levels in plasma were assessed using fluorescence polarization immunoassay (FPIA; Abbott Diagnostics (Abbott AxSym analyzer, Abbott^®^), Chicago, IL, United States). In FPIA method, dithiothreitol (DTT) was employed for thiol liberation, followed by the application of S-adenosyl-homocysteine (SAH) hydrolase to catalyze homocysteine conversion to SAH in the presence of added adenosine. Subsequently, FPIA was executed utilizing a specific monoclonal antibody and a fluoresceinated SAH analog tracer. Total homocysteine concentrations in the FPIA method were computed using the Abbott AxSym^®^ machine, utilizing a pre-stored calibration curve (18).

### 2.3 Vitamin C measurement

Vitamin C was obtained and quantified through isocratic high-performance liquid chromatography with electrochemical detection at 650 mV. Quantitation of peak area was subsequently determined using a standard curve derived from three distinct concentrations of an external standard (0.025, 0.150, and 0.500 mg/dL). The quality assurance and quality control protocols implemented by NHANES adhered to the mandates of the 1988 Clinical Laboratory Improvement Act (19).

### 2.4 Covariates

Covariates encompassed demographic characteristics and health-related behaviors. Demographic attributes comprised age (in years), gender (male vs. female), and Race/ethnicity (Non-Hispanic white, Non-Hispanic black, Mexican American, Other), marital status (unmarried/widowed/divorced vs. Married/living with others), educational attainment (Less than 9th grade, 12th grade, high school grade, some college or AA degree, college graduate or above), ratio of income to poverty [PIR; PIR was divide into three classes: the poor (PIR < 1.3), the middle class (PIR1.3–3.49), and the rich (PIR > 3.5)].The health-related behaviors included smoking (defined as 6 months or more of smoking at least one cigarette a day), alcohol use (During the past year, drank at least one glass of alcohol, equivalent to half a bottle of beer, 125 milliliters of grape wine, fruit wine, or 40 milliliters of white wine), body mass index [BMI = weight (kg)/height (m)^2^], Physical activity [Do you engage in at least half an hour of physical activity (including normal daily activities) during work and/or leisure time every day?], Diabetes (yes vs. no), Cardiovascular disease (yes vs. no), antihypertensive drug use (yes vs. no).

### 2.5 Statistical analysis

Statistical analyses were executed using SPSS 26.0 (IBM Corp., Armonk, NY, United States). Descriptive statistics, including frequencies and percentages [n (%)], were employed to elucidate the demographic characteristics of the participants, whereas continuous data adhering to a normal distribution were expressed as mean ± standard deviation (x ± s), if not adhering to a normal distribution were expressed as median (interquartile rang, IQR). The difference between the two groups should be compared using the rank sum test. The evaluation of internal consistency and reliability involved the calculation of Cronbach’s alpha values. A correlation matrix was established through partial correlation analysis, controlling for covariances. The exploration of influencing factors on blood pressure was conducted using a linear regression model. Confounding adjustments were made for variables associated with homocysteine and Vitamin C. Significance was determined at a 0.05 threshold for all two-sided tests. Mediating effects were assessed utilizing the PROCESS bootstrap methods (20). Major test: the direct effect relationship between homocysteine and blood pressure; mediating effect of Vitamin C; the chain mediating effect of homocysteine and blood pressure. The analysis involved 5,000 bias-corrected bootstrap resamples, and the effect size was assessed using a bias-corrected percentile bootstrap confidence interval (CI). A 95% CI excluding zero indicated statistical significance (21). The determination of the mediated proportion entailed dividing the indirect effect by the total effect.

## 3 Results

### 3.1 Population characteristics

A total of 7,327 participants, comprising 3,594 males and 3,733 females, met the eligibility criteria. The average age was 49.1 years. 21.9% of the participants were found to have hypertension. The average of the homocysteine was 8.1(6.6, 10.2) umol/L. The average of Vitamin C level was 55.1(35.2, 70.4) umol/L. Significant differences between normal and hypertension groups were apparent in all characteristics, with the exception of gender (*p* = 0.331), smoking (*p* = 0.052; Table 1).

**Table 1:** Demographic characteristics of participants (*n* = 7,327).

Variables	Total (*n* = 7,327)	Hypertension group (*n* = 1,607)	Normal group (*n* = 5,720)	χ^2^/t/Z	*p*
Age, (years)	49.1 ± 18.9	62.5 ± 16.0	45.4 ± 18.0	36.82	<0.001
Male, n (%)	3,594(49.1)	803(50.1)	2,791(48.8)	0.94	0.331
Race/ethnicity, n (%)				21.04	<0.001
Non-Hispanic white	1,472(20.1)	270(16.9)	1,202(21.0)		
Non-Hispanic black	217(3.0)	39(2.4)	178(3.1)		
Mexican American	3,915(53.4)	867(54.1)	3,048(53.2)		
Other	1723(23.5)	426(26.6)	1,297(22.7)		
Marital status, n (%)				5.08	0.024
Unmarried/Widowed/Divorced	2,710(37.0)	631(39.4)	2079(36.3)		
Married/living with others	4,617(63.0)	971(60.6)	3,646(63.7)		
Educational attainment, n (%)				100.07	<0.001
Less than 9th grade	943(12.9)	296(18.5)	647(11.3)		
12th grade	1,072(14.6)	245(15.3)	827(14.4)		
High school grade	1788(24.4)	434(27.1)	1,354(23.7)		
Some college or AA degree	2083(28.4)	407(25.4)	1,676(29.3)		
College graduate or above	1,441(19.7)	220(13.7)	1,221(21.3)		
PIR, n (%)				25.36	<0.001
<1.3	1892(25.8)	438(27.3)	1,454(25.4)		
1.3–3.49	2,732(37.3)	658(41.1)	2074(36.2)		
≥3.5	2,703(36.9)	506(31.6)	2,197(38.4)		
Smoking, n (%)	3,593(49.0)	820(51.2)	2,773(48.4)	3.78	0.052
Alcohol use, n (%)	5,119(69.9)	1,040(64.9)	4,079(71.2)	23.82	<0.001
BMI, (kg/m^2^)	28.5(6.3)	29.3(6.7)	28.3(6.1)	5.84	<0.001
Physical activity, n (%)				129.37	<0.001
Yes	2085(28.5)	289(18.0)	1796(31.4)		
No	4,933(67.3)	1,204(75.2)	3,729(65.1)		
Unable to do activity, n (%)	309(4.2)	109(6.8)	200(3.5)		
Diabetes, n (%)	725(9.9)	252(15.7)	473(8.3)	78.31	<0.001
Cardiovascular disease, n (%)	810(11.1)	309(19.3)	501(8.8)	141.35	<0.001
Antihypertensive drug use, n (%)	1751(23.9)	730(45.6)	1,021(17.8)	529.4	<0.001
Homocysteine, (umol/L)	8.1(6.6,10.2)	9.4(7.7,11.7)	7.8(6.3,9.7)	−20.14	<0.001
Vitamin C, (umol/L)	55.1(35.2,70.4)	53.4(31.8,71.0)	55.1(36.3,70.4)	−2.09	<0.036

SD, standard deviation; BMI, body mass index; PIR, ratio of income to poverty.

### 3.2 Correlation analysis

As shown in Table 2, the homocysteine was positively associated with SBP (*r* = 0.24, *p* < 0.001) and DBP (*r* = 0.03, *p* < 0.05), while negatively correlated with Vitamin C (*r* = −0.008, *p* < 0.001). Vitamin C was found to be negatively associated with SBP (*r* = −0.03, *p* < 0.05) and DBP (*r* = 0.11, *p* < 0.001).

**Table 2:** Correlation matrix (*n* = 7,327).

Variables	Homocysteine	Vitamin C	SBP	DBP
Homocysteine	1	-	-	-
Vitamin C	−0.08**	1	-	-
SBP	0.24**	−0.03*	1	-
DBP	0.03*	−0.11**	0.31**	1

*p < 0.05; **p < 0.001; SD, standard deviation.

### 3.3 Linear regression analysis

Table 3 showed the interaction between Vitamin C and homocysteine on SBP/DBP. In unadjusted Model 1, the results indicated a positive correlation between homocysteine and SBP (*β*: 1.03 [0.93, 1.12], *p* < 0.001). Furthermore, homocysteine exhibited a positive correlation with DBP (*β*: 0.08 [0.02, 0.15], *p* = 0.015). Conversely, Vitamin C demonstrated a negative correlation with both SBP (*β*: −0.02 [−0.04, −0.01], *p* < 0.001) and DBP (*β*: −0.05 [−0.06, −0.04], *p* < 0.001). In fully adjusted Model 3, we observed a positive correlation between homocysteine and SBP (*β*: 0.16 [0.01, 0.31], *p* = 0.035). Conversely, Vitamin C exhibited a negative correlation with SBP (*β*: −0.05 [−0.08, −0.02], *p* < 0.001) and DBP (*β*: −0.04 [−0.06, −0.02], *p* < 0.001).

**Table 3:** Linear regression model for interaction between Vitamin C and homocysteine on SBP/DBP (*n* = 7,327).

Variables	Model 1		Model 2		Model 3	
	*p* value	*β* (95%CI)	*p* value	*β* (95%CI)	*p* value	*β* (95%CI)
SBP						
Homocysteine	<0.001	1.03(0.93,1.12)	<0.001	0.23(0.14,0.33)	0.035	0.16(0.01,0.31)
Vitamin C	0.014	−0.02(−0.04,-0.01)	<0.001	−0.04(−0.06,-0.03)	<0.001	−0.05(−0.08,-0.02)
Homocysteine ×Vitamin C	NA	NA	NA	NA	0.674	0.01(−0.01,0.03)
DBP						
Homocysteine	0.015	0.08(0.02,0.15)	0.156	0.05(−0.02,0.13)	0.165	0.08(−0.04,0.20)
Vitamin C	<0.001	−0.05(−0.06,-0.04)	<0.001	−0.05(−0.06,-0.03)	<0.001	−0.04(−0.06,-0.02)
Homocysteine ×Vitamin C	NA	NA	NA	NA	0.437	0.01(−0.02,0.01)

Model l = Vitamin C or homocysteine separately; Model 2 = Model l + covariates (age, gender, race, marital status, educational level, PIR, smoking, alcohol use, physical activity, BMI, diabetes, cardiovascular disease, antihypertensive drug use); Model 3 = Model 2 + interaction between Vitamin C or homocysteine separately; β = βvalue; 95%CI = 95% confidence interval; NA, not apply.

### 3.4 Mediation analysis

Based on the mediation model, the total effect of Vitamin C at homocysteine on SBP was significant (total effect: 0.2138, 95% CI:0.1219–0.3058; *p* < 0.001). The Vitamin C partially mediated the homocysteine–incident SBP association (indirect effect:0.0247, 95% CI:0.0108–0.0455, *p* < 0.001). And the proportion mediated was 11.5% (0.0247/0.2138). However, the mediating effect of Vitamin C between homocysteine and DBP was not statistically significant (Table 4).

**Table 4:** The mediation effect of Vitamin C on the relationship between homocysteine and SBP/DBP (*n* = 7,327)^a^.

Effect types	*Effect*	*SE*	*p* value	Bias-corrected *95%CI*	
				Lower	Upper
SBP					
Total effect	0.2138	0.0469	<0.001	0.1219	0.3058
Direct effects	0.1891	0.0470	<0.001	0.0970	0.2812
Indirect effects	0.0247	0.0090	<0.001	0.0108	0.0455
DBP					
Total effect	0.0716	0.0368	0.0519	−0.0006	0.1438
Direct effects	0.0468	0.0368	0.2034	−0.0253	0.1190
Indirect effects	0.0247	0.0083	<0.001	0.0009	0.0032

SE, standard error; 95%CI, 95% confident interval; ^a^All analysis under controlling for age, gender, race, marital status, educational level, PIR, smoking, alcohol use, physical activity, BMI, diabetes, cardiovascular disease, antihypertensive drug use.

## 4 Discussion

In this study, we investigated the relationship between homocysteine and blood pressure, scrutinizing the mediating influence of Vitamin C on this relationship. We found that higher homocysteine and lower Vitamin C were significantly associated with higher blood pressure. Furthermore, outcomes from the mediation analysis that Vitamin C assumed a partial mediating role in the association between homocysteine and SBP.

We discovered a significant association between elevated homocysteine and the risk of hypertension in our research. The findings from our study are in harmony with a meta-analysis involving 40,173 individuals subjected to Mendelian randomization, indicating a plausible connection between elevated homocysteine levels and an increased risk of hypertension (22). Likewise, Thom et al. (23) showed that every increase of 5 μmol/L in homocysteine resulted in a corresponding increase of 0.5 mmHg and 0.7 mmHg in systolic and diastolic blood pressure. Some fundamental studies have shown that homocysteine can elevate blood pressure through insulin resistance, inducing oxidative stress, antagonizing angiotensin-converting enzyme inhibitors and other mechanisms (24–26). Prior research findings have highlighted the crucial role of the activated form of vitamin B12, namely methylation, serving as an indispensable coenzyme for both homocysteine and methionine. The process of methylation, which involves the conversion of homocysteine to methionine, requires folic acid as a fundamental substrate. Therefore, elevated serum homocysteine levels may not only be attributed to a deficiency in vitamin B12 but also to a deficiency in folic acid (27). In clinical settings, the concurrent administration of folic acid and vitamin B12 has demonstrated a substantial reduction in serum homocysteine levels among individuals with H-type hypertension. Furthermore, a notable decrease in both systolic and diastolic blood pressure levels was observed in these patients (28, 29). In real life, folic acid and vitamin B12 should be supplemented to reduce homocysteine.

This study showed that homocysteine was negatively associated with Vitamin C. Krajcovicova-Kudlackova et al. (30) found a negative correlation between plasma homocysteine and vitamin C in healthy adults, which is consistent with our results. A comprehensive study involving 5,812 participants demonstrated an inverse association between plasma homocysteine and vitamin C intake (31). Homocysteine, a sulfur-containing amino acid, arises through the intermediary metabolism of methionine (9). The prooxidant effects induced by thiol compounds are predominantly ascribed to the generation of reactive species, encompassing superoxide and hydrogen peroxide. The auto-oxidation of homocysteine in the presence of transition metal ions leads to the generation of hydrogen peroxide. Homocysteine is linked to endothelial dysfunction, a process mediated through oxidant stress mechanisms, and can be mitigated by antioxidants. Vitamin C is effective scavengers of reactive oxygen species (16). This may be the reason for the negative correlation between homocysteine and vitamin C.

Our study revealed a noteworthy correlation between Vitamin C and blood pressure. A prospective investigation study show that robust association was observed between elevated vitamin C concentrations and lower blood pressure levels., which consistent with our findings (32). In a meta-analysis comprising 29 short-term (<1 year) and predominantly small-sized randomized controlled trials, vitamin C supplementation was observed to moderately reduce blood pressure (33). Ran et al. (34) scrutinized 11 cross-sectional studies and 7 case–control studies conducted between 1990 and 2017. The findings revealed that individuals with hypertension exhibited notably lower serum vitamin C levels compared to their normotensive counterparts. Furthermore, the authors identified a substantial inverse correlation was identified between serum vitamin C concentration and SBP and DBP. Research indicates that vitamin C enhances the synthesis of PGE1 and Prostacyclin (PGI2), both of which exert potent vasodilator effects. Moreover, both PGE1 and PGI2 augment endothelial nitric oxide generation by endothelial cells. Therefore, vitamin C, through its capacity to enhance the synthesis of PGE1, PGI2, and NO, exerts cytoprotective, anti-mutagenic, vasodilator, and platelet anti-aggregator actions, potentially elucidating the beneficial effects of vitamin C on hypertension (14). Prior research has indicated that the development of hypertension is concomitant with endothelial dysfunction characterized by intravascular oxidative stress (35). Vitamin C could potentially exert an influence on blood pressure reduction by fostering vascular relaxation and augmenting nitric oxide production. Diminished nitric oxide levels may result in arterial stiffness and constriction, thereby contributing to the potential elevation of blood pressure (36).

One interesting aspect of this study is that the association between homocysteine and blood pressure was mediated by Vitamin C. Homocysteine is linked to endothelial dysfunction, a condition mediated through oxidant stress mechanisms and susceptible to inhibition by antioxidants. Vitamin C serves as a powerful antioxidant, mitigating oxidative stress and promoting improved endothelial function. It achieves this by scavenging intracellular superoxide, subsequently activating smooth muscle guanylyl cyclase and endothelial nitric oxide synthase. This cascade of events may contribute to a potential reduction in blood pressure (37). The interaction homocysteine on Vitamin C instigate alterations in vascular function, consequently contributing to the development of hypertension.

## 5 Strengths and limitations

A key strength inherent in this study resides in its utilization of data derived from a nationally representative sample. Noteworthy strengths encompass a substantial sample size and an extensive compilation of nutritional information. In order to make the results more reliable, our analysis included adjustment for multiple confounders. We delved not only into the correlation between homocysteine and blood pressure but also scrutinized its underlying mechanisms using mediation analysis.

This study has several potential limitations. Firstly, the information was self-reported by participants, which may have recall bias. Secondly, we were unable to estimate the impact of changes in homocysteine and Vitamin C during the blood pressure because NHANES 2003–2006 collected homocysteine and Vitamin C information at baseline only. Ultimately, it is acknowledged that plasma vitamin C in serum serves as an indicator of a relatively short-term supply, potentially not carrying the same significance as intracellular levels in portraying the status of these vitamins.

## 6 Conclusion

Higher homocysteine levels are associated with a higher risk of hypertension. Vitamin C plays a significant role as a mediator in the association between homocysteine and blood pressure. These findings suggest that lowering homocysteine levels and increasing vitamin C may help reduce the risk of hypertension.

## Data availability statement

The original contributions presented in the study are included in the article/supplementary material, further inquiries can be directed to the corresponding authors.

## Ethics statement

The studies involving humans were approved by the Research Ethics Review Board of the National Center for Health Statistics. The studies were conducted in accordance with the local legislation and institutional requirements. The participants provided their written informed consent to participate in this study.

## Author contributions

PW: Conceptualization, Data curation, Formal analysis, Investigation, Methodology, Software, Supervision, Validation, Visualization, Writing – original draft. JM: Conceptualization, Data curation, Formal analysis, Investigation, Methodology, Software, Supervision, Validation, Visualization, Writing – original draft. SY: Data curation, Methodology, Writing – original draft. HW: Data curation, Methodology, Writing – original draft. XM: Data curation, Writing – original draft. DC: Investigation, Writing – original draft. SJ: Investigation, Supervision, Validation, Writing – review & editing. NY: Investigation, Supervision, Validation, Writing – review & editing.

## Footnotes

^1^ https://www.cdc.gov/nchs/nhanes/index.htm
